# Arsenic Toxicity: Molecular Targets and Therapeutic Agents

**DOI:** 10.3390/biom10020235

**Published:** 2020-02-04

**Authors:** Valeria M. Nurchi, Aleksandra Buha Djordjevic, Guido Crisponi, Jan Alexander, Geir Bjørklund, Jan Aaseth

**Affiliations:** 1 Department of Life and Environmental Sciences, University of Cagliari, Cittadella Universitaria, 09124 Monserrato-Cagliari, Italy; crisponi@unica.it; 2 Department of Toxicology “Akademik Danilo Soldatović”, Faculty of Pharmacy, University of Belgrade, 11000 Belgrade, Serbia; aleksandra.buha@pharmacy.bg.ac.rs; 3 Norwegian Institute of Public Health, 0213 Oslo, Norway; jan.alexander@fhi.no; 4 Council for Nutritional and Environmental Medicine, 8610 Mo i Rana, Norway; bjorklund@conem.org; 5 Research Department, Innlandet Hospital Trust, 2380 Brumunddal, Norway; 6 IM Sechenov First Moscow State Medical University (Sechenov University), Bolshaya Pirogovskaya St., 19-1, 119146 Moscow, Russia

**Keywords:** arsenic, drinking water, arsenic poisoning, lipoic acid, BAL, DMPS

## Abstract

High arsenic (As) levels in food and drinking water, or under some occupational conditions, can precipitate chronic toxicity and in some cases cancer. Millions of people are exposed to unacceptable amounts of As through drinking water and food. Highly exposed individuals may develop acute, subacute, or chronic signs of poisoning, characterized by skin lesions, cardiovascular symptoms, and in some cases, multi-organ failure. Inorganic arsenite(III) and organic arsenicals with the general formula R-As^2+^ are bound tightly to thiol groups, particularly to vicinal dithiols such as dihydrolipoic acid (DHLA), which together with some seleno-enzymes constitute vulnerable targets for the toxic action of As. In addition, R-As^2+^-compounds have even higher affinity to selenol groups, e.g., in thioredoxin reductase that also possesses a thiol group vicinal to the selenol. Inhibition of this and other ROS scavenging seleno-enzymes explain the oxidative stress associated with arsenic poisoning. The development of chelating agents, such as the dithiols BAL (dimercaptopropanol), DMPS (dimercapto-propanesulfonate) and DMSA (dimercaptosuccinic acid), took advantage of the fact that As had high affinity towards vicinal dithiols. Primary prevention by reducing exposure of the millions of people exposed to unacceptable As levels should be the prioritized strategy. However, in acute and subacute and even some cases with chronic As poisonings chelation treatment with therapeutic dithiols, in particular DMPS appears promising as regards alleviation of symptoms. In acute cases, initial treatment with BAL combined with DMPS should be considered.

## 1. Introduction

The element arsenic (As) belongs to Group 15 in the periodic system. Additionally, it is chemically classified as a metalloid. It occurs in many minerals in the Earth’s crust, often together with other metals and sulfur. In recent years China, together with Russia and Morocco, have been the top producers of As [1]. However, nowadays most As refinement operations have been phased out in the USA and Europe, due to environmental concerns.

Until recently, several compounds of As were used as pesticides and herbicides [2]. Due to its antifungal properties, chromated copper arsenate was used to treat and preserve wood. However, the use of this As compound in consumer products was banned in 2004 in the USA as well as in the European Union due to mounting evidence of As toxicity [3]. Spraying of fruit trees with methylated arsenates, as well as with other arsenicals, have also been extensively used until recently, due to their insecticidal properties. However, the use of arsenicals in agricultural activities has been phasing out in the Western world from about 2013. Nowadays, the US Agency for Toxic Substances and Disease Registry places As as number one on their Priority List of Hazardous Substances [4]. The International Agency for Research on Cancer (IARC) classifies arsenic (As) and inorganic arsenic (iAs) compounds in Group 1, carcinogenic to humans [5].

In their hazard assessment, The Joint FAO/WHO Expert Committee on Food Additives (JECFA) [6] modeled dose-response data from recent studies on lung and urinary tract cancer and established a benchmark dose for 0.5% elevated lung cancer incidence above background, with 95% confidence limit, for a daily dose between 3.0 and 5.0 µg As/kg body weight (mainly iAs). They selected the lowest dose of 3.0 µg As/kg body weight per day as a risk assessment reference point. In different countries in Europe and Asia as well as in the USA, it has been reported that mean dietary iAs exposure ranges from 0.1 to 3.0 µg As/kg body weight per day, illustrating that As in food and drinking water may constitute a public health concern [7]. In 2014, the WHO held an advisory conference to confirm limits for rice of 200–300 µg/kg [8].

In 2006, the US Environmental Protection Agency (EPA) set 10 µg/L as the maximum allowed concentration of As in drinking water [9]. The Occupational Safety and Health Administration has set a permissible exposure limit (PEL) as a time-weighted average (TWA) of 0.01 mg/m^3^. The National Institute for Occupational Safety and Health (NIOSH) has set a recommended exposure limit (REL) of 0.002 mg As/m^3^ for 15-min constant exposure [10].

The biochemistry of As is similar to other elements found in Group 15 of the periodic table, in particular phosphorous, and to some extent also nitrogen. Thus, arsenate (AsO_4_^3−^), the dominating As species in seawater, occurs together with phosphate (PO_4_^3−^), and these two compounds are indistinguishable to marine algae, explaining their high AsO_4_^3−^ uptake. During changing conditions of salinity, aquatic organisms synthesize the nitrogen-containing compound glycine betaine to maintain the osmotic balance. However, due to structural mimicry As can replace nitrogen in this synthesis [11], which may explain the high arsenobetaine concentrations in the marine nutrition chain.

The abundant occurrence of organoarsenicals, in nature as in the laboratory, is a result of the high electronegativity of As, viz. 2.18 on the Pauling scale, which is a little lower than that of carbon (2.55), sulfur (2.58) and selenium (2.55). Consequently, As has a high tendency, compared with other metals, to be involved in covalent bonding to carbon, as well as to sulfur and selenium [12].

Before the developments of modern antibiotics and cytostatic agents, different organic as well as iAs compounds were used as pharmaceuticals, for instance, Salvarsan (arsphenamine) for syphilis [13] and arsenic trioxide for cancer [14]. In treatment of psoriasis, the As-containing Fowler’s solution was recommended, but later it was found in a dose-related manner to increase the risk of skin cancer [15], Still. As(III) trioxide is approved as a treatment in cases of acute promyelocytic leukemia, but only when conventional regimens are not effective [16].

Arsenic has four oxidation states, i.e., −3, 0, +3, and +5. Under aerobic conditions, the latter is the dominant oxidation state [17]. This review focuses on the +3 and +5 oxidation states of As. Today both of these series of compounds have worldwide toxicological relevance, which motivated to elaborate the present overview. In the present work we aim to give a narrative review of environmental sources of As exposure, health hazards, molecular targets of toxicity and therapeutic measures in acute and chronic As toxicity.

Literature published after the year 2000 has been searched in Pubmed, Medline and Google scholar by making use of the search keyword As combined with a second keyword, either environment, toxicity, targets or therapeutics. In addition, references to some earlier papers are included to illustrate historical perspectives. The article is focused on inorganic arsenic compounds (iAs), and the symbol As refers to the element arsenic not further specified.

## 2. Sources of Exposure

### 2.1. Arsenic in Drinking Water

Worldwide an estimated 200 million people are exposed to As in drinking water above the WHO recommended guideline of 10 µg/L [18]. The majority lives in southern Asia, but also populations living in other areas are affected. Arsenic in ground water and drinking water occurs as iAs, but is in chemical analyses usually determined as total As.

**Figure 1:**
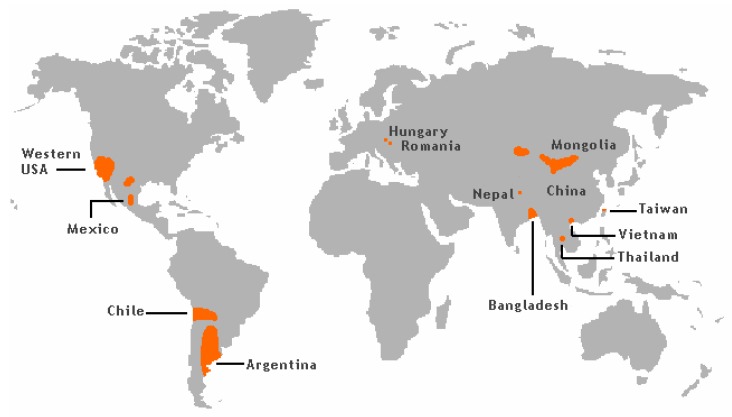
Arsenic risk areas around the world. From [20], Wikimedia Commons, the free media repository.

The groundwater levels of As has caused significant exposure and frequent As poisoning, as is reported i.a. from West Bengal and Bangladesh (Figure 1) [19].

More than 50 million people in these regions drink groundwater containing As levels above 50 µg/L [21]. It is known that As occurring in sediments in some areas of the world can be released into the groundwater. Southeast Asian districts and countries, including Cambodia, Vietnam, China, and Taiwan have groundwater with high As content due to their geological environments [22].

In the USA, groundwater in the southwest regions frequently contains significant amounts of As (Figure 1). Thus, it is estimated that the As values in about 16% of wells in New Mexico exceed 10 µg/L, most of this As contamination being focused in the Middle Rio Grande Basin [23,24]. In Arizona, values in the range 10–210 µg/L have been reported for well water [25].

High levels of As in ground and surface water related to mining activities have been reported from Peru [26]. In a district in Northern Chile the As content in drinking water exceeded 100 µg/L until 1979 when an As-removal plant was built. In fact, after 2004 levels of As in drinking water in the same region had dropped to about 10 µg/L [27]. At different sampling sites in Northern Argentina, the measured concentrations of As in drinking water were as high as about 2000 µg As/L [28]. These high levels in Chile and Argentina are explained by geological characteristics related to the Andes mountain range.

Recent studies conducted in Serbia, showed substantially elevated levels of As when compared with the limit of 10 µg/L. Determined levels of As in investigated water samples from the public water supply system of the Northern region of Serbia were more than ten-fold higher than the recommendation and even reached levels above 300 µg/L [29]. These findings can be explained by geological characteristics of the Northern region of Serbia belonging to the Pannonian Basin (Hungary, Romania, Croatia and Serbia) known to contain elevated naturally occurring As. It has been estimated that nearly 500,000 people are exposed to levels above the recommended limit, making this region the largest affected area in Europe [30].

### 2.2. Arsenic in Food

For individuals who are neither occupationally exposed to As nor via drinking water, the most important source of As is food [31]. Among food of earthbound origin, some cereals, such as rice have As concentrations which are sometimes as high as 0.4 mg/kg dry weight [6]. The main As species in rice and other plants irrigated with As-containing water or grown on As rich soil is iAs [7,32].

In contrast to terrestrial organisms that mainly contain inorganic As, most of As in marine organisms are organoarsenicals. In particular, in organisms from the upper part of the marine food chain, arsenobetaine is the prevalent arsenical compound [33]. However, in marine algae, arsenosugars occur as the most significant arsenicals [34], and these are prevalent also in shellfish [33]. Arsenolipids, also synthesized in algae in place of phosphate, occur in the lipid phase of marine organisms and are present in various types of seafood, including cod liver oil and tuna [35,36]. Marine fish, as well as other kinds of marine seafood, contain As concentrations up to 100 mg As/kg [32]. While in marine organisms arsenobetaine is a more common arsenical [33], fresh-water organisms also contain As as arsenobetaine, but in much lower concentrations. Lower-ranking organisms contain a larger fraction bound to sugars and in various other low molecular weight compounds including sulfur-containing molecules.

### 2.3. Occupational Exposure

Occupational exposure to As compounds is another important source of exposure. Occupational exposure in some cases can even lead to As poisoning, especially in industries that are using inorganic As, or in workers using other toxic arsenicals. These include wood preservation, vineyard spraying, nonferrous metal alloys, glass production, and production of electronic semiconductors [37]. Inorganic As (iAs) is also emitted from the smelter industries, such as As or copper smelters, resulting in both occupational and environmental exposure associated with increased lung cancer risk among workers and residents in the vicinity of a smelter [38,39].

## 3. Health Effects of Arsenic Exposure

Acute arsenic poisoning is associated initially with nausea, vomiting, abdominal pain, and severe diarrhea [40]. Encephalopathy and peripheral neuropathy may occur. Paresthesia in the limbs is a frequent symptom of iAs exposure, which in some cases may develop into widespread polyneuropathy [41].

Prolonged iAs exposure affects the skin, particularly with localization at skin folds. This can give rise to changes in pigmentation, hyperkeratosis and over time, development of skin cancer, e.g., in the hands. Upon such exposure As is a well-documented human carcinogen affecting numerous organs [5]. Significantly increased risks of lung, urinary tract as well as skin cancer are reported at levels in drinking water around and above 50 µg/L [42]. Analyses of exposure to (As) in many epidemiological studies suggest that there might be a small elevation of the risk to develop bladder cancer when the As levels in drinking water are as low as 10 µg/L [43], although a recent meta-analysis revealed no increase in risk at this level [44]. JECFA noted, however, that in epidemiological studies in that low dose-range the risk probably would be too low to be detected, due to interferences from confounding factors like cigarette smoking. In Northern Chile, as in Southeast Asian districts, epidemiological evidence has shown a dose-dependent association between chronic As exposure and the different cancer forms [45], An increased risk of bladder as well as lung cancer persisted for at least three decades after a high exposure had ended [46].

Other sensitive endpoints of prolonged exposure are peripheral neuropathy, cardiovascular disease, and in exposed children neurobehavioral effects [47]. Some research suggests that exposure to environmental As might be a risk factor in children for the development of autism spectrum disorder. However, these findings are inconclusive [48,49]. In experimental studies exposure to As disturbs the neurotransmitter metabolism. Arsenic disrupts the glutamate-induced release of gliotransmitters, causing changes in the neuronal function [50]. In addition, iAs exposure may impair the transport of glutamate [51]. In rats, iAs exposure significantly impacted brain cholinergic receptors [52]. Furthermore, in cells, the interplay between dopamine and iAs increased the neurotoxic effects on the dopaminergic neurons [53].

Arsenic exposure can also precipitate type 2 diabetes mellitus in susceptible cases [54]. Long-term iAs exposure induces increased oxidative stress, which may explain deteriorations in structures and functions of the cardiovascular system. Furthermore, by increasing the tendency of platelet aggregation, As can aggravate atherosclerosis. Endothelial nitric oxide synthase is inactivated by As, which causes reduced nitric oxide production in the vascular bed [55]. Arsenic exposures may also upregulate the expression of interleukin-1, tumor necrosis factor-α, vascular endothelial growth factor, and vascular cell adhesion molecule, and thereby causing endothelial dysfunction and aggravating cardiovascular pathology [56]. Furthermore, epidemiological observation studies in humans showed an association between As exposure and impaired reproductive function in males [57].

## 4. The Metabolism and Mechanisms of Toxicity

Arsenic undergoes in vivo several complex metabolic conversions. Both As and its metabolites interact with extra- and intracellular macromolecules, in particular, those containing vicinal thiols, but the mechanism of action might vary with respect to chemical form of As [7,58]. Methylation of iAs has been considered as a part of a detoxification pathway for many years. However, newer research indicates that intermediary metabolites, in particular, monomethylarsonous acid (MMA-III), but also dimethylarsonous acid (DMA-III), are reactive and toxic [59]. Although MMA-III and DMA-III are usually quickly oxidized to the less toxic pentavalent species, the intermediates in the iAs methylation pathways can cause toxic effects, including DNA-damage [7]. Indeed, a higher primary iAs methylation activity has in epidemiological studies been associated with an increased risk of skin lesions including skin cancer [6].

Research has identified a monomethyl arsonic acid reductase (MMA-V reductase) that function as a catalyst when As(V), DMA(V), and MMA(V) are reduced to the more toxic trivalent species [60], presumably through reactions depending on the presence of reduced glutathione [61]. Recently, in a study in mice, it has been found that oral DMA-V in significant amounts is reduced to DMA-III in the intestine and liver as first-pass effects, and bonded extensively to tissues and red blood cells [62].

Both the peripheral and the central nervous systems may be affected by As neurotoxicity [63]. In particular, As exposure significantly affects the glial component of the central nervous system [64]. The neurotoxic effects caused by As exposure seems to be oxidative stress-mediated [65]. Mitochondria is the major target for As neurotoxicity [66]. Arsenic inhibits the complexes I, II, and IV of the electron transport chain, which elevate mitochondrial production of reactive oxygen species. In turn, this mitochondrial disturbance may lead to microglial cell apoptosis [67]. Microglia is more sensitive to As (III) toxicity than to As (V) [68]. Brain morphology in rodents may be significantly affected by As exposure, which results in neuronal degeneration, gliosis, and disruption of the blood-brain barrier [69]. Arsenic may impair neurite outgrowth [70]. Chronic As exposure in rats causes neuronal apoptosis in the hippocampus, and cognitive impairments, like spatial memory impairment [71,72,73]. In mice exposed to As, As-induced alteration in the metabolic pathway of arachidonic acid may be a factor in the mediation of neuronal damage and inflammatory response [74]. In astrocytes, sub-toxic doses of monomethylarsonous acid increase the pro-inflammatory cytokines gene expression significantly [75]. Neuronal apoptosis is induced both by dimethylarsinic acid and iAs [76]. In particular, one class of arsenolipids, arsenohydrocarbons, is toxic to human neurons in vitro and is able to cross an in vitro brain barrier model [77,78,79]. Hence, this class of compounds might have a potential for neurodevelopmental toxicity.

The acute toxicity of As is ascribed to the strong affinity of As (III) for SH-groups. It is now well known that endogenous thiols can be occupied by As compounds, such as in some cofactors (i.a. lipoic acid) or cysteine residues that are essential constituents in the active sites of crucial enzymes. Thus, early studies disclosed that As (III)-compounds block the function of the sulfur-containing pyruvate dehydrogenase enzyme complex, which is crucial for the conversion of pyruvate to acetyl-CoA that enters the citric acid cycle. This vulnerable enzyme complex is essential for mitochondrial functions [80]. Experimentally, some of the mitochondrial effects appear to be reversed by administration of the cofactor α-lipoic acid [81]. Additionally, As (V) competes with phosphate, thus further inhibiting or blocking the mitochondrial respiration and the ATP synthesis. These metabolic interferences may lead to death from multi-organ failure.

It has been documented that As(III)-compounds inhibit cytosolic SH-enzymes such as glutathione reductase [82]. Other important targets are selenol groups in, e.g., the seleno-enzymes glutathione peroxidase and thioredoxin reductase, the latter also containing a thiol group vicinal to the selenol group [16,82]. Through these interactions, As appears to impair scavenging of intracellular hydrogen peroxide (H_2_O_2_) production, which can react further to highly reactive and short-lived hydroxyl radicals.

Usually As-III-compounds are considered to represent the most toxic and carcinogenic species, and mechanisms of actions including chromosomal effects are discussed in a recent review [83]. However, it should be noted that DMA-V can induce bladder cancer in rats and lung tumors in mice [6]. Research has linked long-term As exposure to epigenetic changes, which might also cause heritable gene expression changes [84]. These changes include histone modification, RNA interference, and DNA methylation [85]. For instance, excessive levels of As cause critical DNA hypermethylation via tumor protein p53, which increase the carcinogenesis risk. Other important mechanisms of carcinogenicity are inhibition of various DNA repair systems as well as interference with redox regulation and ROS production [86,87,88,89]. The organic arsenical arsenobetaine, the dominating species in seafood, is excreted unchanged and considered to be of low toxicity and is not classified as carcinogenic. The possible toxicities of arsenosugars and arsenolipids are insufficiently studied, but these species may split off DMA in the intestine [90] where, as mentioned above, it might be reduced to the bioactive trivalent DMA-III.

## 5. Treatment of Arsenic Poisoning

The classic antidote against acute arsenic poisoning, BAL (British anti-Lewite, dimercaptopropanol), was originally developed as a war gas antidote for Lewisite (dichlorovinyl arsine). BAL is a dithiol compound, which competes successfully with endogenous SH-groups, e.g., with the sulfur groups of the pyruvate dehydrogenase cofactor α-lipoate for As in cases of arsenical poisonings, thereby enhancing As elimination from the body.

However, due to the rather high toxicity of BAL, and the necessity of frequent and inconvenient intramuscular administrations [91,92], the clinical use of this drug is currently restricted to only the initial treatment for few days after acute As intoxications [93]. Water-soluble and less toxic derivatives of BAL have been developed for therapeutic use, viz. DMSA (dimercaptosuccinic acid) and DMPS (dimercaptopropane sulfonate) which was in regular clinical use in China [94] and the former Soviet Union [95] early after 1950. Several decades had to pass after their original introduction [96,97] before Western clinicians fully realized their value. Today, treatment of acute and also some severe cases of chronic As poisoning makes use of DMPS as chelating antidote [98]. In acute life-threatening poisonings, it is recommended to support vital functions and perform chelation therapy as fast as possible. The therapy implies BAL i.m. in doses calculated as 5 mg/kg and a maximum of 300 mg i.m. four times daily during the first days, combined with DMPS i.v. to provide a more efficient therapeutic approach than obtained with monotherapy in the initial stage [98,99]. It is reasonable to presume that BAL can act as a shuttling agent for intra- to the extracellular transfer of As in emergency cases, the extracellular metal is then picked up by circulating DMPS with the formed As-DMPS-chelate being rapidly excreted via urine. Successful treatment with DMPS has been described in different case reports [100,101]. After the initial high-dosed therapy, the chelation regimen is usually switched to oral monotherapy with DMPS. In acute cases of massive arsenic trioxide poisoning supplementation of the chelator combination with gastric rinsing and forced alkaline diuresis, has been recommended [102]. If renal failure occurs, hemodialysis should be initiated and combined with chelation. The present recommendations have been precipitated from a recent extensive review of the available database of case reports and animal experiments on chelation therapy in As poisonings [103].

The efficacy of DMPS in chronic As toxicity was observed in a placebo-controlled study of patients exposed to As-contaminated drinking water. DMPS was given orally to 11 patients for three weeks with inter-current chelator-free weeks, and ten patients received a placebo. Significant improvement in symptoms of neuropathy and lung symptoms was found in the DMPS group compared to the placebo group [104]. In contrast, only negligible improvement has been observed after monotherapy with DMSA [104,105]. To the knowledge of the authors, combinations of BAL or DMPS with lipoic acid (LA) or its reduced form (DHLA) have yet not been tried clinically in such poisonings. With regard to the carcinogenic effect of prolonged As exposure, it has been hypothesized that an adequate or supra-nutritional status of selenium may exert a protective action [106]. This hypothesis is supported by some epidemiological and experimental studies [107,108], but further research is needed.

## 6. Chemical Features of BAL, DMSA, and DMPS and Their As(III)-Chelates

Many of the toxic compounds and metabolites of inorganic and organic arsenicals can be described by the general formula, R-As^2+^, which explains that vicinal dithiols constitute particularly vulnerable targets. Vicinal dithiols such as DHLA, BAL, DMPS, and DMSA are also the most efficient chelating antidotes.

### 6.1. Protonation Constants

We report the acidic properties of the chelating agents DHLA, BAL, DMSA, and DMPS, to obtain insight into the chemical form and behavior of the parent molecules in vivo, i.e., in blood with pH 7.4 and urine with pH usually about 6. The protonation constants are of particular importance since they determine the biological properties of a drug, such as its solubility, absorption, cell penetration, and bioavailability. Furthermore, protonation constants are of primary importance also in determining the speciation of the complexes formed with trivalent arsenic. Table 1 reports selected protonation constants of these ligands (those concerning the SH-groups are marked in red) together with their structure, the used acronyms, the formulae, and the molecular weights. It appears that the protonation constants (log K_1_) of the SH-groups of BAL, DMSA, DMPS and DHLA are in the range 10.6–12, while log K_2_ is about two units lower, ranging from 8.6 to 9.9, the differences among the various ligands depending on the charge of the molecule and on the distance between the mercapto groups.

Lipoic acid is also included in Table 1 since this enzymatic cofactor is considered a primary target of the As (III) toxicity when the metalloid blocks the pyruvate dehydrogenase complex.

### 6.2. Chemical Stabilities of the As-Chelates

Available literature reports only few stoichiometric data on the complex formation equilibria between the thiol chelating agents and As (III) compounds. However, a number of papers describe the principal features of complexes studied with different techniques, briefly summarized in the following.

**Figure 2:**
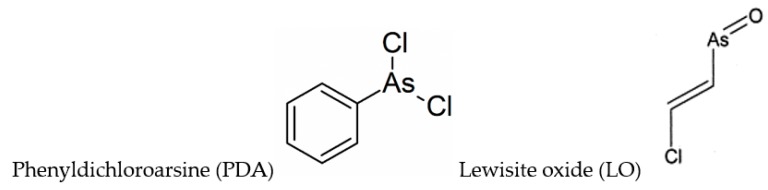
Chemical formulae of phenyldichloroarsine (PDA) and Lewisite oxide (LO).

A paper by Dill et al. [113] reports one- and two-dimensional (homonuclear and heteronuclear) NMR studies on BAL and its phenyldichloroarsine (PDA) adduct, where PDA is a Lewisite analog (Figure 2). PDA appears to react with BAL such that in the complex, the hydroxymethyl group is *anti* to the phenyl ring, and to a less extent a *syn* structure was observed. A successive paper by the same authors [114] took into consideration the structures of DMSA and DMPS adducts with PDA, and Lewisite (trans-2-chlorovinylarsine) oxide (LO) (Figure 2).

The 1:1 adducts were synthesized and characterized by one- and two-dimensional NMR spectroscopy. All the formed complexes were five-membered heteroatomic ring systems (chelates), with different solubility properties (Figure S1). The results indicated that the functional groups of the chelating agent influenced stereochemical aspects of adduct formation [114].

Adams et al. presented an X-Ray and spectroscopic investigation on the interaction of several arylarsenic dichlorides with BAL and DMSA [115]. In the single crystal of the complex between CH_3_C_6_H_4_AsCl_2_ and BAL the arsenic was found in a distorted-square-pyramidal coordination geometry (Table S1), with one of the sulfur atoms on each of the chelating ligands considerably further away from the arsenic than the other. The five-membered ring presents with an *envelope* configuration where the hydroxymethyl group is located on a hinge of the envelope *anti* to the phenyl group of the arsenic. Although no single crystals of the DMSA adducts were suitable for X-ray diffraction, the IR spectra of the complexes clearly showed both the OH groups of the ligand as well as its C=O stretch, and the stretches due to substituted phenyl groups. The ^13^C NMR spectrum was also highly indicative of arsenic coordination.

**Figure 3:**
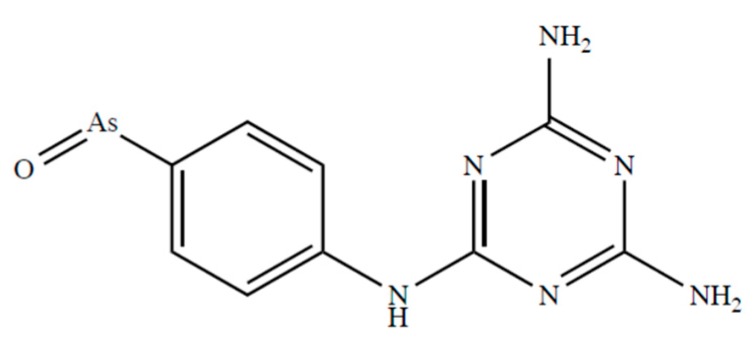
Chemical formulae of melarsen oxide.

Fairlamb et al. [116] report that D,L-dihydrolipoamide and DHLA form stable complexes with melarsen oxide (Figure 3) with complex formation constants of 5.47 × 10^9^ and 4.51 × 10^9^ M^−1^, respectively.

**Figure 4:**
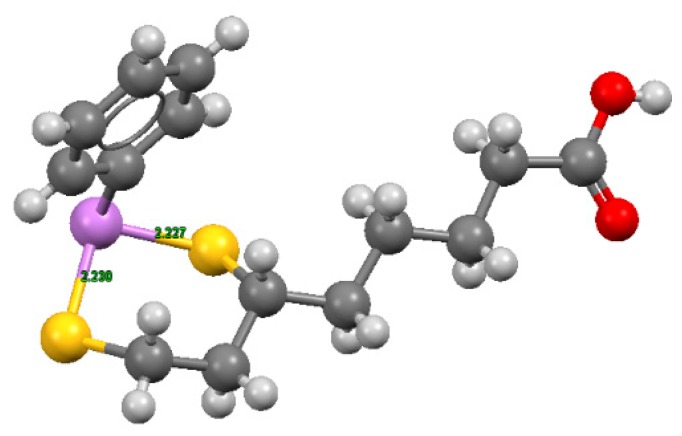
Structures of phenylarsenic with DHLA, where hydrogen is shown in light grey, carbon in grey, sulfur in yellow, oxygen in red, and As (III) in violet. The coordinates were obtained from the Cambridge Structural Database (NIDKAM), and the image was created using Mercury3.5.

These complexes, characterized by six-membered cyclic dithioarsenite rings, are 10-fold less stable than the complexes with five-membered rings found with BAL (79.30 × 10^9^ M^−1^) and DMSA (45.00 × 10^9^ M^−1^) by Fairlamb et al. [117]. The solid-state structure of the complex between PhAs (III) and DHLA shown in Figure 4, was reported by von Döllen and Strasdeit [118].

Spuches et al. [119] used near-UV absorption spectroscopy and isothermal titration calorimetry to quantify the stability and thermodynamics of formation of arsenite and monomethylarsenite (MMA) complexes with DMSA, dithiothreitol (DTT), and DHLA. They found that MMA forms chelates with the dithiols somewhat more stable than those of arsenite.

Cavanillas et al. [120] made use of a new methodology that combines voltammetry, ITC, ESI-MS, and several chemometric tools to the study of As(III) complexes with DMSA and DMPS, obtaining estimates of the stoichiometries of the complexes formed (ML_2_, with the appearance of only minor amounts of ML-species), and approximately similar As(III)-complex formation constants for the two agents (log β_2_ 9.2 and 9.8 for DMSA and DMPS, respectively).

Harper and Bayse [121] used density functional theory (DFT) and solvent-assisted proton exchange (SAPE) to model the chelation of the As in Lewisite by the vicinal thiols of BAL. They concluded, “the low barriers for lewisite detoxification by BAL and the greater stability of the chelation complexes of small dithiols are consistent with the rapid reversal of toxicity demonstrated in previously reported animal models”.

## 7. Discussion and Conclusions

Humans are exposed to As compounds from many sources, including occupational and environmental sources, which include drinking water and food items. Exposure may range from acutely toxic to various levels of long-term or life-long exposure. High As levels in food or drinking water, or under some occupational conditions, can in some cases precipitate chronic poisoning and in some cases cancer. Individuals subjected to high or long-term exposure may develop acute, subacute, or chronic signs of poisoning, characterized by skin lesions, cardiovascular diseases, and/or neurological symptoms. Pentavalent organic As found in marine species appears to be less toxic than trivalent inorganic and methylated species.

The reviewed analytical results support the hypotheses that the endogenous vicinal dithiol DHLA, and the therapeutic agents BAL and DMPS, have a particularly high affinity for arsenical compounds. The relative affinity of selenol groups for arsenicals may be even higher, but determinations of the chemical stabilities of these chelates are sparse or missing.

Apparently, both inorganic arsenite (III) and R-As^2+^-compounds are bound tightly to thiol groups, which constitute vulnerable targets for the toxic action of As. Selenol groups, e.g., in seleno-enzymes and particularly in conjunction with thiol groups as in thioredoxin reductase, are anticipated to have an even higher affinity to R-As^2+^-compounds, explaining the oxidative stress associated with arsenic toxicity. The strong general affinity to thiols and selenols may explain the many targets of arsenicals and a wide range of adverse health effects. Among therapeutic agents, the most efficient chelators are the dithiols BAL and DMPS, both of which with particularly high affinity to R-As^2+^-compound.

Whereas primary prevention aiming to reduce environmental exposure to toxic inorganic As and organic As from drinking water and food for the many millions of people with unacceptable exposure to As should be the prioritized approach, therapeutic intervention may be needed in cases of acute, subacute, and even chronic As poisonings. Here chelation with DMPS, which can be administered orally or intravenously, appears promising as regards alleviation of symptoms. In severe acute cases, a combination of DMPS intravenously with BAL intramuscularly can be used in the first days of the treatment.

## Figures and Tables

**Table 1:** Protonation constants of BAL, DMSA, DMPS, lipoic acid (LA), and dihydrolipoic acid (DHLA).

Structure	Name	Formula	MW	log K_1_	log K_2_	log K_3_	log K_4_
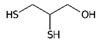	BAL	C_3_H_8_OS_2_	124.23	[109] 10.62	8.65		
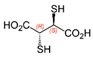	DMSA	C_4_H_6_O_4_S_2_	182.22	[110] 12.05	9.65	3.43	2.71
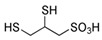	DMPS	C_3_H_8_O_3_S_3_	188.28	[109] 11.38	8.69		
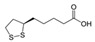	LA	C_8_H_14_O_2_S_2_	206.34	[111] 4.704			
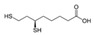	DHLA	C_8_H_16_O_2_S_2_	208.34	[112] 11.02	9.86	4.73	

The protonation constants related to the SH-groups are marked in red.

## Supplementary Materials

The following are available online at https://www.mdpi.com/2218-273X/10/2/235/s1, Figure S1. Molecular formulae of the 1:1 adducts proposed in reference [114] based on NMR results formed between A) *trans*-2-chlorovinylarsine oxide or lewisite oxide (LO) and DHLA; B) LO and DMSA; C) LO and DMPS; D) PDA and DMSA; E) PDA and DMPS, Table S1. Structures of As(III) with different ligands bearing mercapto groups, where hydrogen is shown in light grey, carbon in grey, nitrogen in blue, sulfur in yellow, oxygen in red, and As(III) in violet. The coordinates were obtained from the Cambridge Structural Database, and the image was created using Mercury3.5.
