# Supplementary material for: Arsenic Toxicity: Molecular Targets and Therapeutic Agents
Source: Biomolecules. 2020 Feb 4;10(2):235. doi: 10.3390/biom10020235 (PMC7072575; doi:10.3390/biom10020235)
Supplement: Supplementary file 1 [file biomolecules-10-00235-s001.pdf]

biomolecules

MDPI

Supplementary Materials

Arsenic Toxicity: Molecular Targets and Therapeutic Agents

Valeria M. Nurchi [1,*], Aleksandra Buha Djordjevic [2], Guido Crisponi [1], Jan Alexander [3], Geir Bjørklund [4] and Jan Aaseth [5,6,*]

[1] Department of Life and Environmental Sciences, University of Cagliari, Italy2
[2] Department of Toxicology "Akademik Danilo Soldatović", Faculty of Pharmacy, University of Belgrade, Serbia; Orcid: 000-0002-6942-7040
[3] Norwegian Institute of Public Health, Oslo, Norway, Orcid: 0000-0002-6381-5720
[4] Council for Nutritional and Environmental Medicine, Mo i Rana, Norway
[5] Research Department, Innlandet Hospital Trust, Brumunddal, Norway
[6] IM Sechenov First Moscow State Medical University (Sechenov University), Bolshaya Pirogovskaya St., 19-1, 119146, Moscow, Russia
* Correspondence: nurchi@unica.it (V.M.N.); jaol-aas@online.no (J.A.)

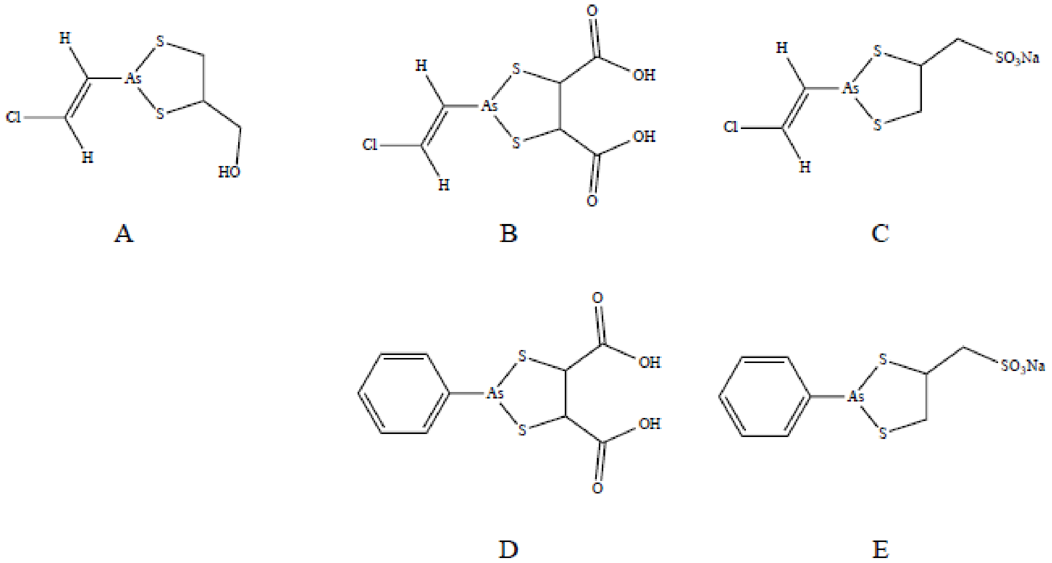

Figure S1. Molecular formulae of the 1:1 adducts proposed in the paper "O'Connor, R.J.; McGown, E.L.; Dill, K.; Hallowell, S.F. Two-dimensional NMR studies of arsenical-sulfhydryl adducts. *Magn. Reson. Chem.* **1989**, *27*, 669–675" based on NMR results formed between (**A**) lewisite oxide (LO) and DHLA; (**B**) LO and DMSA; (**C**) LO and DMPS ; (**D**) Phenyldichloroarsine (PDA) and DMSA; (**E**) PDA and DMPS.

Table S1. Structures of As(III) with different ligands bearing mercapto groups, where hydrogen is shown in light grey, carbon in grey, nitrogen in blue, sulfur in yellow, oxygen in red, and As(III) in violet. The coordinates were obtained from the Cambridge Structural Database, and the image was created using Mercury3.5.

CCDC-Refcode; Reference	As(III) Structures	Complex	Ligand
VETDAZ<br>E. Adams, D. Jeter, A.W. Cordes, J.W. Kolis, Inorg. Chem., (1990) 29, 1500-1503	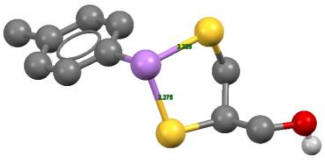	As(III)-L	BAL
NEJQOJ<br>T.A. Shaikh, R.C. Bakus II, S. Parkin, D.A. Atwood, J. Organomet. Chem., (2006) 691, 1825-1833	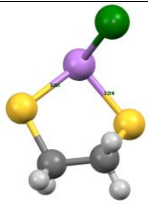	As(III)-L-I	$SHCH_2CH_2SH$
NEJQUP<br>T.A. Shaikh, R.C. Bakus II, S. Parkin, D.A. Atwood, J. Organomet. Chem., (2006) 691, 1825-1833	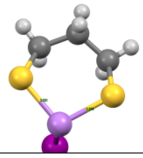	As(III)-L-Cl	$SHCH_2CH_2CH_2SH$
JUKNUV<br>T.A. Shaikh, R.C. Bakus II, S. Parkin, D.A. Atwood, J. Organomet. Chem., (2006) 691, 1825-1833	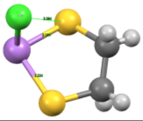	As(III)-L-Cl	$SHCH_2CH_2SH$
NIDKAM<br>A.von Dollen, H.Strasdeit, Eu. J. Inorg. Chem, (1998) 61-66,	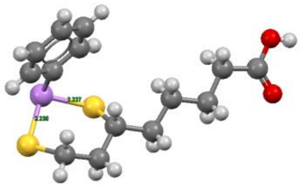	As(III)Ph-L	DHLA

© 2020 by the authors. Licensee MDPI, Basel, Switzerland. This article is an open access article distributed under the terms and conditions of the Creative Commons Attribution (CC BY) license (http://creativecommons.org/licenses/by/4.0/).
